# Scaling Relationships of Maximal Gape in Two Species of Large Invasive Snakes, Brown Treesnakes and Burmese Pythons, and Implications for Maximal Prey Size

**DOI:** 10.1093/iob/obac033

**Published:** 2022-08-25

**Authors:** Bruce C Jayne, Abigail L Bamberger, Douglas R Mader, Ian A Bartoszek

**Affiliations:** Department of Biological Sciences, University of Cincinnati, Cincinnati, OH 45221-0006, USA; Department of Biological Sciences, University of Cincinnati, Cincinnati, OH 45221-0006, USA; Tropical Veterinary Services, Big Pine Key, FL 33043, USA; Conservancy of Southwest Florida, Naples, FL 34102, USA

## Abstract

Snakes are a phylogenetically diverse (> 3500 species) clade of gape-limited predators that consume diverse prey and have considerable ontogenetic and interspecific variation in size, but empirical data on maximal gape are very limited. To test how overall size predicts gape, we quantified the scaling relationships between maximal gape, overall size, and several cranial dimensions for a wide range of sizes (mass 8–64,100 g) for two large, invasive snake species: Burmese pythons (*Python molorus bivittatus*) and brown treesnakes (*Boiga irregularis*). Although skull size scaled with negative allometry relative to overall size, isometry and positive allometry commonly occurred for other measurements. For similar snout-vent lengths (SVL), the maximal gape areas of Burmese pythons were approximately 4–6 times greater than those of brown treesnakes, mainly as a result of having a significantly larger relative contribution to gape by the intermandibular soft tissues (43% vs. 17%). In both snake species and for all types of prey, the scaling relationships predicted that relative prey mass (RPM) at maximal gape decreased precipitously with increased overall snake size. For a given SVL or mass, the predicted maximal values of RPM of the Burmese pythons exceeded those of brown treesnakes for all prey types, and predicted values of RPM were usually least for chickens, greatest for limbed reptiles and intermediate for mammals. The pythons we studied are noteworthy for having large overall size and gape that is large even after correcting for overall size, both of which could facilitate some large individuals (SVL = 5 m) exploiting very large vertebrate prey (e.g., deer > 50 kg). Although brown treesnakes had longer quadrate bones, Burmese pythons had larger absolute and larger relative gape as a combined result of larger overall size, larger relative head size, and most importantly, greater stretch of the soft tissues.

## Introduction

Size has widespread and profound effects on nearly all aspects of the structure and function of animals including those involved in predation (Calder 1984; Schmidt-Nielsen 1984; Bonner 2006), and size commonly requires a variety of metrics such as length, area, and mass (proportional to volume) to be fully understood. In addition to size often varying among different species, large ontogenetic changes in different metrics of size (Y and X) occur and are often well described by the equation Y = bX^a^ (equivalent to logY = logb + a*logX), where a is the scaling exponent (Huxley and Teissier 1936). Geometrically similar animals have isometric scaling where all lengths (L) scale with other lengths with an exponent of 1, but positive and negative allometry refer to scaling exponents more or less than 1, respectively (Huxley and Teissier 1936). Thus, with geometric similarity, areas and volumes (masses) scale with L^2^ and L^3^, respectively; hence, area and length scale with mass^2/3^ and mass^1/3^, respectively (Table 1). This results in a more rapid increase of mass relative to length and area that is especially important for understanding how the metabolic demands of predators change with their overall size.

**Table 1:** Least squares regression statistics (±95%CL) for the scaling equations of snake morphology.

Variables			Burmese python (*n *= 43)				Brown treesnake (*n* = 19)			
ind	dep	exp	obs	slope	intercept	*R* ^2^	obs	slope	intercept	*R* ^2^
SVL	mass	3	+	3.260 ± 0.151	**−3.847 **± 0.349	0.979	+	3.398 ± 0.283	**−**4.684 ± 0.560	0.974
SVL	Gdiam	1	**−**	0.870 ± 0.067	**−**1.055 ± 0.154	0.944	=	**1.009 **± 0.113	**−**1.608 ± 0.225	0.954
SVL	Garea	2	**−**	1.739 ± 0.134	**−**2.215 ± 0.308	0.944	=	**2.017 **± 0.227	**−**3.320 ± 0.449	0.954
SVL	SKL	1	**−**	0.732 ± 0.036	**−0.942 **± 0.084	0.976	**−**	0.668 ± 0.069	**−**0.983 ± 0.136	0.961
SVL	SKW	1	**−**	0.783 ± 0.079	**−1.475 **± 0.181	0.908	**−**	0.753 ± 0.136	**−**1.511 ± 0.270	0.889
SVL	QL	1	**−**	0.735 ± 0.038	**−**1.509 ± 0.088	0.973	+	**1.192 **± 0.067	**−**2.364 ± 0.132	0.988
SVL	LJDL	1	**−**	0.750 ± 0.042	**−**0.925 ± 0.096	0.970	**−**	**0.891 **± 0.070	**−**1.354 ± 0.138	0.977
SKL	Garea	2	+	2.366 ± 0.155	0.030 ± 1.117	0.959	+	**3.000 **± 0.215	**−**0.348 ± 0.077	0.981
ALL	Garea	2	+	2.304 ± 0.141	**−1.094 **± 1.178	0.963	=	2.142 ± 0.176	**−**1.273 ± 0.162	0.975
mass	Garea	0.67	**−**	0.531 ± 0.036	**−0.155 **± 0.132	0.957	**−**	0.588 ± 0.061	**−**0.529 ± 0.130	0.961
mass	SKL	0.33	**−**	**0.222 **± 0.011	**−**0.070 ± 0.040	0.977	**−**	0.196 ± 0.014	**−**0.060 ± 0.030	0.980
SKL	QL	1	=	0.998 ± 0.036	**−**0.561 ± 0.027	0.987	+	**1.730 **± 0.163	**−**0.593 ± 0.059	0.967
SKL	LJDL	1	=	1.022 ± 0.037	0.043 ± 0.028	0.987	+	**1.318 **± 0.059	**−**0.038 ± 0.021	0.992
SKW	QL	1	**−**	0.871 ± 0.075	**−**0.104 ± 0.028	0.931	+	**1.425 **± 0.243	0.024 ± 0.040	0.900
SKW	LJDL	1	**−**	0.892 ± 0.075	0.509 ± 0.028	0.934	=	**1.082 **± 0.165	0.432 ± 0.027	0.919

Independent and dependent variables are indicated by ind and dep, respectively. The slopes expected from geometric similarity are indicated by exp. Observed slopes (obs) that conformed to isometry (based on 95% CL) or had negative or positive allometry are indicated by = ,− and +, respectively. All *P*-values for the test of the overall significance of the regression (slope not equal to 0) were less than 10^–8^. Abbreviations: SVL, snout-vent length; Gdiam, maximal gape diameter; Garea, maximal gape area; SKL, skull length; SKW skull width; QL quadrate length; LJDL length of the entire lower jaw; ALL = SKW + 2QL + 2LJDL. Units of distance, area and mass are cm, cm^2^ and g, respectively, and all values were log_10_ transformed. If the slopes differed significantly between the two species in an ANCOVA (with the independent variable as the covariate), bold typeface indicates the slope of the species with a greater value. If the slopes were homogenous, then the species with the significantly greater mean adjusted for the covariate is indicated by boldface type for its intercept. See Table S2 for ANCOVA results.

The sizes of both predator and prey are important for understanding feeding ecology because the energetic demands of predators are proportional to their size, and the energy consumed is proportional to meal or prey size. Consequently, RPM (RPM = prey mass/predator mass) is a widely used and useful metric, in part because of its implications for how frequently a predator feeds (Greene 1983). Because snakes swallow their prey whole, the maximal size of their mouth opening (gape diameter (Gdiam) or gape area (Garea)) imposes an anatomical limit on prey size. However, when different size snakes consume prey that are a constant fraction of their maximal gape (e.g., prey area/Garea), RPM will only be constant for the seemingly unlikely scenario when both snakes and their prey have identical scaling exponents for mass as a function of the dimensions that are relevant to gape. Furthermore, for a given gape and cross-sectional area of prey, different prey shapes can affect values of RPM (Greene 1983; Voris and Voris 1983). Despite this clear importance of integrating scaling relationships of gape with additional measures of the size and shape of both predator and prey, such data are only available for four species of snakes, all of which have extremely specialized diets of only crustaceans (Jayne et al. 2018; Gripshover and Jayne 2021).

Snakes are predators with diverse diets as the more than 3500 extant species have considerable ecological, morphological, and phylogenetic diversity. Furthermore, large gape is a key evolutionary innovation that was long used to define a diverse group (>3000 species) of “macrostomate” snakes, many of which consume vertebrate rather than invertebrate prey (Colston et al. 2010; Hsiang et al. 2015; Grundler and Rabosky 2021). However, macrostomates are probably paraphyletic (Cundall and Greene 2000; Cundall 2019; Zaher et al. 2019; Caldwell 2020). Presently, maximal gape has only been measured directly using rather small snakes (most lengths <1 m) belonging to nine species, eight of which are caenophidians (Hampton and Moon 2013; Hampton and Kalmus 2014; Hampton 2018a; Jayne et al. 2018; Gripshover and Jayne 2021). Consequently, the current lack of quantitative gape measurements has impeded precisely defining what “large” gape and macrostomy are as these are fundamentally quantitative traits.

The lack of empirical measurements of gape has also impeded understanding the relative importance of different structures for enhancing the gape of snakes, which can be affected by (1) the dimensions of bones, (2) the orientation of bones, and (3) the distension relevant soft tissues. Decades ago a seminal paper on organismal performance used a schematic figure to show how the dimensions of different bones could affect gape (performance) and thus be acted upon by natural selection (Arnold 1983). The importance of distension of soft tissues for contributing to gape has also been emphasized repeatedly, particularly for macrostomate snakes (Close and Cundall 2014). However, despite numerous discussions of the potential determinants of gape, empirical data on the relative contributions of different jaw bones and the soft tissue between the jaws have only recently been determined for two species of snakes with very limited phylogenetic diversity (Gripshover and Jayne 2021). Importantly, variation in the contribution of the soft tissues to gape will reduce the predictive value of variation in skeletal dimensions.

**Fig. 1:**
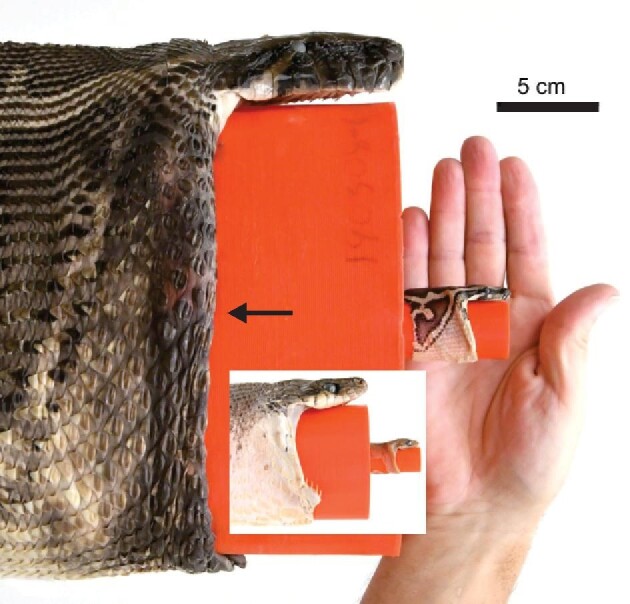
Range of sizes used to determine maximal gape. The inset shows brown treesnakes (SVL = 40,177 cm; gape = 1.2, 5.7 cm), whereas the Burmese pythons (SVL = 61,397 cm; gape = 2.8, 22 cm) are in the background. All images are to the same scale. The arrow indicates the distal end of the lower jaw in the large python. The small python contained substantial yolk. At maximal gape, the circumferential distance between scales exceeded the width of scales in the neck, whereas the scales at rest completely covered the regions of skin in between them.

The paraphyletic group formerly known as “henophidia” consists of the pythons, boas, and their relatives that are not included in either the scoleophidia or caenophidia, and this assemblage is of special interest for understanding the evolution of large gape because of diverging earlier relative to caenophidians. Furthermore, unlike the earlier diverging lineages (scolephidians) that eat small invertebrate prey, several henophidian lineages eat mainly vertebrate prey (Grundler and Rabosky 2021). Henophidians also have many species larger than the largest caenophidian, and they are all huge compared to scolecophidians. However, the only non-caenophidian gape data are for the pipe snake, *Cylindrophis ruffus* (Hampton 2018a), and this rather small species is often not considered to be a macrostomate. By contrast, large pythons are macrostomates that are renowned for attacking and eating large prey, in some rare cases including humans (Headland and Greene 2011; Natusch et al. 2021). Whether this ability to eat extremely large prey is mainly a result of large overall size or also large gape for their overall size is presently unclear because of the lack of data for the scaling relationships for maximal gape. Nonetheless, the combination of large interspecific and ontogenetic variation in the overall size of these snakes (Fig. 1) makes them ideal for studying and gaining insights into the scaling relationships between the sizes of predators and their prey.

Our two study species, Burmese pythons (*Python molorus bivittatus*, Pythonidae) and brown treesnakes (*Boiga irregularis*, Colubridae, Caenophidia), are phylogenetically distant, and these generalist predators have highly invasive populations in south Florida, and Guam, respectively (Rodda et al. 1999b; Taillie et al. 2021). Burmese pythons attain huge sizes and are one of only four species of extant snakes with maximal mass > 100 kg (Barker et al. 2012; Rivas 2020), nearly 1000 times greater than hatchling mass (Secor 1995; Pittman and Bartoszek 2021). Brown treesnakes (maximal total length ∼ 3 m) are also reasonably large for a colubrid snake, with large adults as much as 200 times heavier than neonates (Rodda et al. 1999a; Feldman and Meiri 2013). Brown treesnakes and many arboreal snakes are also slender compared to many terrestrial species (Lillywhite and Henderson 1993; Rodda et al. 1999a; Pizzatto et al. 2007). Besides the utility of these two species with highly variable size (Fig. 1) for gaining basic insights into the scaling of gape and its effects on the potential prey size, studying these two invasive species can provide practical information regarding their potential ecological impact.

We tested whether our two study species differed as follows. First, we determined the scaling relationships between maximum gape and different metrics of size. This allowed us to test whether scaling exponents conformed to theoretical null hypotheses based on geometric similarity (isometry) and whether the two study species had different scaling relationships. Second, we quantified the relative importance of skeletal dimensions and the soft tissue between the lower jaws for contributing to maximal gape, in part to test alternative hypotheses that species with larger gape attain this primarily from greater distension of the soft tissues or from longer lengths of the relevant bones. Third, we determined how the three-dimensional orientations of major skeletal structures changed from rest posture to maximal gape. Finally, we determined the potential consequences of different prey shape for RPM by integrating scaling data for gape and diverse vertebrate prey, in part to test between alternatives for snakes of equal length for which (1) a slender species attains greater RPM than a stouter (heavier) species because both species have similar gape and (2) a stout species maintains parity in RPM with a slender species as a result of having greater gape.

## Materials and methods

### Sample

For both study species, we attempted to obtain as large of a size range as possible, and our samples of both species included large adults and snakes near hatchling size (Fig. 1). For example, the two smallest pythons still contained substantial yolk in their abdominal cavity. All procedures were in compliance with the Institutional Animal Care and Use Committee of the University of Cincinnati (protocol number 07–01–08–01).

All 19 brown treesnakes used in the study were collected in Guam (US Fish and Wildlife Service permit MA214902). The ranges of snout-vent length (SVL) and mass for males (*n* = 7) and females (*n* = 12) were 65–184 cm and 24–1138 g, and 40–153 cm and 9–740 g, respectively. The two largest females and the three largest males were long-term captives after being captured as adults, whereas all other individuals were euthanized shortly after capture.

We obtained a total of 43 Burmese pythons (*P.molorus bivittatus*). Six individuals (SVL 97–154 cm) were born and raised in captivity. Rearing snakes in captivity can affect their cranial morphology (Ryerson 2020). However, in our sample of Burmese pythons, the residual values of gape predicted from SVL were both positive and negative and they did not appear to vary in any systematic way compared to wild-caught snakes (Table S1). We collected 37 individuals in southern Florida, USA (Florida Wildlife Commission permit EXOT-18–50, National Park Service permit EVER-2018-SCI-0070). The ranges of SVL and mass for males (*n* = 17) and females (*n* = 26) were 61–287 cm and 100–18,400 g, and 61–435 cm and 100–63,500 g, respectively.

### Measurements of snake morphology

Immediately after euthanizing the snakes, we measured their mass and SVL. We measured gape of all brown treesnakes and 27 pythons immediately after euthanasia, but 16 of the pythons were frozen from 3 days to 3 months before measuring gape. To prevent problems associated with freeze drying in these specimens, they were placed in sealed plastic bags filled with water prior to freezing. To increase the ease of measuring gape and reduce the space required to store specimens, we cut the neck at a distance of approximately two skull lengths posterior to the skull. To measure Gdiam in the brown treesnakes and the pythons, we used the same overall procedures as in Jayne et al. (2018) and Gripshover and Jayne (2021), respectively. For both species as successively large probes were inserted, once the cylindrical portion of the probe was large enough to create appreciable resistance, we pulled the head along the probe by holding its posterior margin and alternately moving contralateral sides to simulate the movements used by snakes to swallow prey. After measuring gape, we used 10% formalin to fix the specimens with a cylindrical spacer in the mouth that had a diameter equal to that of maximal gape (Fig. 1). The cylindrical spacer remained in each specimen for all subsequent procedures. The specimens were subsequently soaked in water and stored in 70% ethanol.

The main portion of all probes was a cylinder, but the shapes of the ends of the probes initially inserted into the mouths of the two study species differed. The probes used for the brown treesnakes were machined out of a solid piece of hard (Delrin®) plastic and had a straight bevel at a 45° angle relative to the long axis of the cylinder, whereas the probes for the pythons were 3-D printed with PLA plastic and had a hemispherical end. The incremental increases in cylinder diameter between successively larger probes were 1, 2, and 3 mm for probe diameters ≤ 20, 20–30, ≥30 mm, respectively, for the Delrin® probes and 1, 2, 3, 4, 5, 10, and 20 mm for probes with diameters of ≤ 22, 22–44, 44–68, 68–80, 80–110,110–200, and ≥200 mm, respectively, for the PLA probes.

**Fig. 2:**
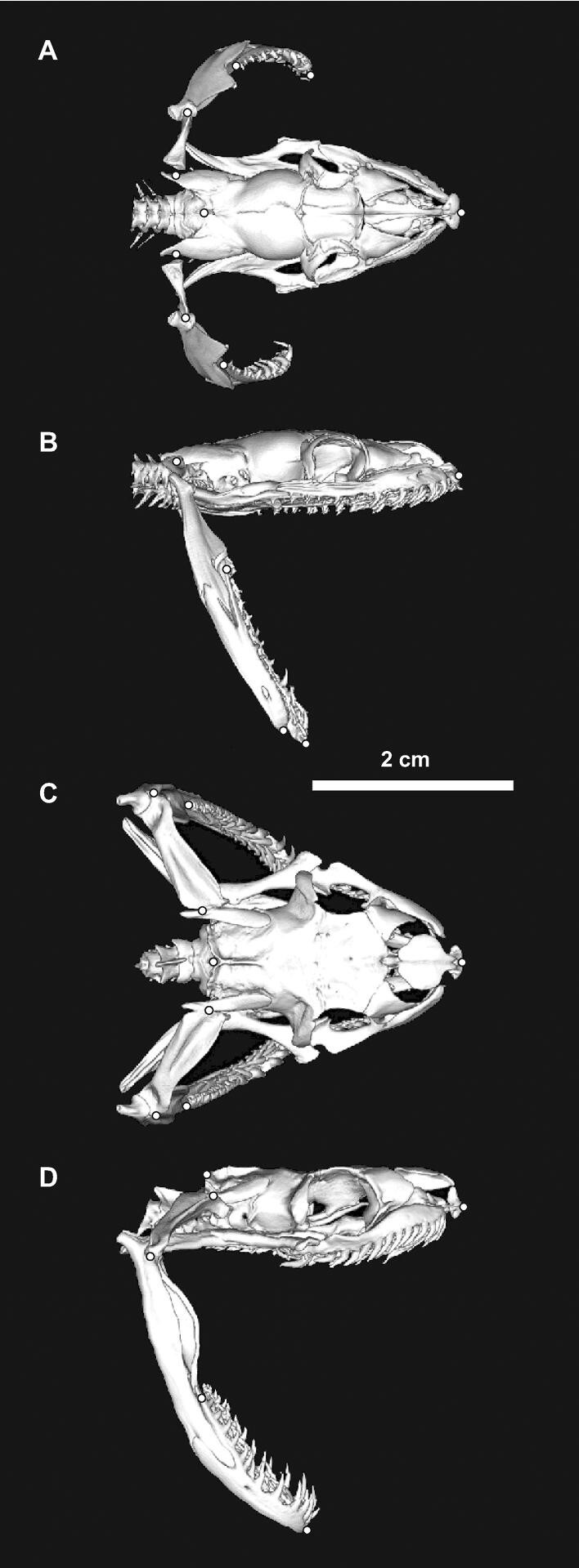
CT scans showing the landmarks (dots) used for morphological measurements. (**A**, **B**) Burmese python (SVL = 61 cm). (**C**, **D**) Brown treesnake (SVL = 130 cm). Both specimens had a maximal Gdiam of 2.8 cm. In the hatchling python, the proximal quadrate did contact the supratemporal bone, but the cartilaginous end of the quadrate is not visible in this rendering. Skull length (SKL) was the distance along the mid-dorsal line from the snout to the dorso-posterior margin of the parietal bones, whereas skull width (SKW) was the transverse distance between the centers of the joints between the quadrate and supratemporal bones.

**Fig. 3:**
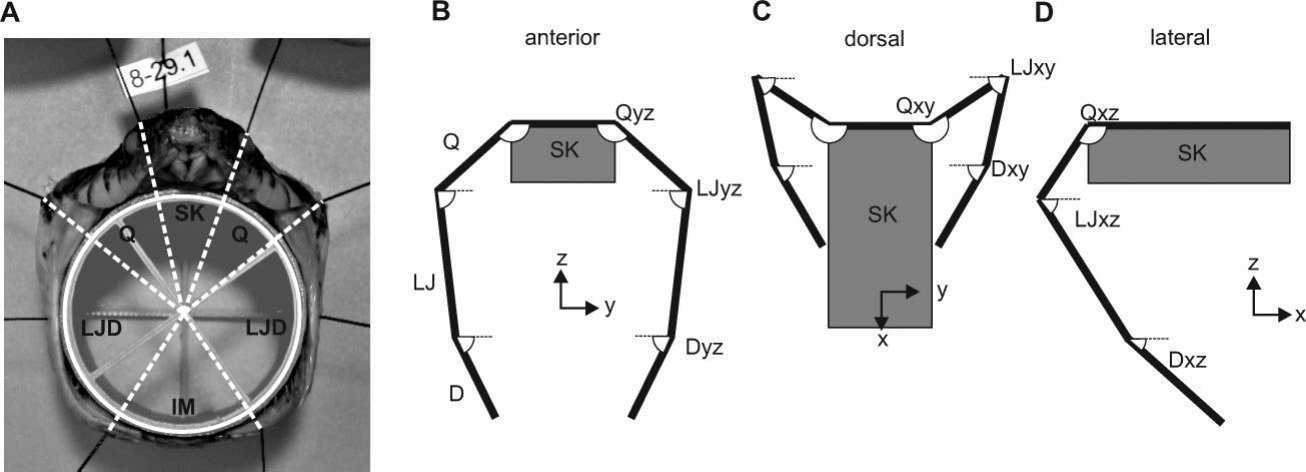
Methods for determining contributions to maximal Garea and orientation of major cranial structures. (**A**) An anterior view of a brown treesnake showing how contributions to gape were determined from pins located at the anatomical landmarks shown in Fig. 2. Garea was partitioned into portions arising from the: skull (SK), quadrate (Q), lower jaw including the dentary (LJD), and intermandibular soft tissues between the tips of the lower jaw (IM). (**B**–**D**) Schematic diagrams showing the orientation of axes and conventions for determining the two-dimensional orientations of major structures in three orthogonal planes. The quadrate, proximal lower jaw, and dentary are abbreviated by Q, LJ, and D, respectively, and for the angles of each structure, the two lowercase letters indicate the plane containing the angle. Angles of 90° indicated that the structure pointed straight down in the anterior (**B**) and lateral (**D**) views, whereas a 90° angle in the dorsal view (**C**) indicated that the structure pointed straight forward. The thick lines in the schematic figures illustrate the stick figures used to summarize the mean orientations and locations of the cranial structures in Fig. 7.

We used the following procedures to determine the sizes of different structures and their contributions to maximal Garea and three-dimensional orientation. We removed sufficient skin and muscle to expose skeletal landmarks that permitted us to use micrometer calipers to measure the length (SKL) and width (SKW) of the skull and the lengths of quadrate (QL) and the lower jaw (LJDL) from its joint with the quadrate to its distal tip (Fig. 2). Pins marked the superficial centers of the proximal and distal joints of the quadrate and the locations of the most posterior tooth of the dentary bone and the distal tip of the dentary bone (Fig. 3A). We inserted 3D printed structures resembling spoked wheels at both ends of the cylindrical spacer (Fig. 3A) to facilitate aligning all specimens to obtain anterior-view photographs. For each species, we used a subset of six individuals preserved at maximal gape and two individuals preserved at rest to obtain three-dimensional coordinates of the skeletal landmarks. These coordinates were obtained from additional photographs that were perpendicular to the longitudinal axis.

To determine the contributions of different structures to gape, we imported all anterior-view photographs into CorelDraw X5. We then constructed a circle with its center aligned with the intersection of the spokes of the cylindrical spacer (Fig. 3A) and manipulated the nodes of arcs along on the circumference of the circle to obtain a readout of the angle of each arc that was used to partition Garea. We partitioned the area of the circle overlapping the cylindrical spacer into four portions, (expressed as percentages of the circular area and calculated by dividing arc angles by 360°) based on (1) the width of the skull (SK) between the landmarks at the quadrate-supratemporal joint, (2) the lengths of the quadrates (Q), (3) the overall lengths of the lower jaws (LJD), and (4) the soft tissues of the intermandibular region (IM) (Fig. 3A). The dentary bone overlapped extensively with the more proximal part of the lower jaw (Fig. 2). Consequently, we partitioned gape using the entire length of the lower jaw rather than using the separate lengths of the proximal lower jaw and dentary bone as in some other analyses.

We also constructed rectangles in CorelDraw for additional orthogonal pairs of photographs to determine the three-dimensional coordinates of the landmarks needed to determine the orientations of the quadrate (Q), proximal lower jaw (LJ), and dentary (D) in three orthogonal anatomical planes (Fig. 3B–D), for which the *x*-axis was parallel to the longitudinal anatomical axis. Fig. 3 shows the conventions used for measuring and labeling all these two-dimensional angles in the anterior, dorsal, and lateral views (yz, xy, and xz planes, respectively). For example, Qyz > 90° indicates that the quadrate in the anterior view had its distal portion lateral to its proximal portion (Fig. 3B).

### Measurements of potential prey morphology

Many vertebrate prey have deformable bodies with nearly circular cross-sectional shapes when swallowed by snakes (Close and Cundall 2012). Hence, we quantified the circular cross-sectional areas of diverse potential prey species using a series of 3D printed funnels with a conical mouth that was 30° relative to the long axis of the funnel stem. The funnels had cylindrical (parallel-sided) stems with lengths that exceeded snout-to-rump length of prey, and the internal diameters of the stems differed by the same amounts as the cylindrical PLA probes used to measure gape.

We obtained frozen domestic rats (*Rattus norvegicus domestica*), rabbits (*Oryctolagus cuniculus domesticus*), chickens (*Gallus gallus domesticus*), raccoons (*Procyon lotor*), and common iguanas (*Iguana iguana*) from commercial sources. After thawing and weighing the specimens, we placed them head-first into the vertically oriented funnel and shook it so that the prey slid through. We used successively smaller funnels until the prey no long slid through the stem of the funnel. We estimated prey cross-sectional area from a circle with a diameter equal to the mean internal diameter of the stems of the last two funnels that were used.

We made similar measurements using live, sedated, captive American alligators (*Alligator mississippiensis*) at an alligator farm in south Florida. We sedated the alligators by injecting Telazol (tiletamine/zolazepam) (Tilozan®, Dechra, Overland Park, KS, USA) into the triceps muscles using 5 or 4 mg/kg for animals less than or greater than 2 kg, respectively. Induction and recovery times were approximately 5 and 45 min, respectively. We gently placed the head of the sedated alligator into a horizontally oriented measuring funnel and then pulled the alligator through the stem of the funnel. The alligators were then monitored for 24 h before being placed back into their normal housing area.

For a total of nine dead white-tailed deer from south Florida (*Odocoileus virginianus seminolus*), we estimated the cross-sectional area from an ellipse with axes defined by the height and width of the chest. The smallest deer was recovered after being recently ingested by a python, whereas the remaining measurements of deer were performed by wildlife biologists working for the Florida Fish and Wildlife Conservation Commission.

### Data analysis

All lengths (cm), areas (cm^2^), and masses (g) were log_10_ transformed before using Microsoft Excel to perform linear ordinary least squares (Kilmer and Rodriguez 2016) regression analyses and to calculate the 95% CL of the slope and intercept of the regression. For all regressions, the test of overall significance for all regressions was whether or not the slope differed significantly from 0. To test whether the calculated slopes differed significantly from those expected from geometric similarity (isometry), we examined whether or not the value expected from isometry (Table 1, column 3) was within the 95% CL of the calculated slope for the regression. To test whether the two species had significantly scaling regressions within each row in Table 1, we used Systat version 9 and the procedures described in (Wilkinson 1992) for performing an analysis of covariance (ANCOVA) with the independent variables in column 1 of Table 1 as the covariate. If the assumption of homogeneity of slopes was met, we proceeded to use an ANCOVA to test for whether the adjusted mean of the dependent variable of one species was significantly different from that of the other species. Details of the ANCOVA results for snake and prey morpholgy are in Tables S2 and S3, respectively. If the slopes of two species were significantly different, then we simply used visual observation of plotted data to discern additional trends regarding whether values of the dependent variable of one species were consistently greater or less than those of the other species. To test for some additional differences between species (e.g., Table 2), we used two sample *t*-tests. Our criterion for statistical significance was 0.05, unless stated otherwise. All mean values are given ± SE.

**Table 2:** Comparisons of mean angles (±SE) of the quadrate and lower jaw bones for snakes at rest and maximal gape.

	Brown treesnake (BTS)		Burmese python (BP)		BTS vs. BP at max gape	
angle	max gape (*n* = 6)	Rest (*n* = 2)	max gape (*n* = 6)	Rest (*n *= 2)	2-tail t (df = 10)	*P*
Dyz	64.1 ± 2.5	**−**36.9 ± 0.8	**101.9 **± 2.3	1.1 ± 0.9	**−**11.11	<0.001
LJyz	91.5 ± 1.7	28.1 ± 0.2	**123.5 **± 1.5	11.7 ± 0.0	**−**14.01	<0.001
Qyz	136.5 ± 1.3	129.7 ± 3.6	139.8 ± 1.5	128.0 ± 2.0	**−**1.70	0.12
Dxy	42.7 ± 5.6	79.3 ± 0.3	**197.3 **± 7.5	72.5 ± 0.4	**−**16.50	<0.001
Ljxy	94.6 ± 4.6	75.3 ± 2.4	**149.2 **± 3.4	78.1 ± 0.8	**−**9.54	<0.001
Qxy	**200.0 **± 2.8	231.6 ± 1.3	174.5 ± 2.2	198.5 ± 4.4	7.20	<0.001
Dxz	65.6 ± 2.9	**−**7.9 ± 0.0	**88.1 **± 1.1	1.3 ± 0.2	**−**7.31	<0.001
LJxz	70.5 ± 0.4	8.4 ± 1.4	68.1 ± 3.1	1.7 ± 0.1	0.77	0.46
Qxz	**110.9 **± 3.5	135.9 ± 2.4	84.2 ± 2.8	105.5 ± 5.1	5.94	<0.001

See Fig. 3 for the conventions used to determine angles in the transverse (yz), frontal (xy), and sagittal (xz) planes, and see Fig. 7 for a graphical summary of these values. All values are in degrees. D, LJ, and Q are the dentary, proximal lower jaw, and quadrate, respectively. For the comparisons of angles of BTS versus BP at maximum gape, when a significant difference occurred between species, the greater mean value is indicated in bold.

## Results

### Scaling relationships of snake anatomy

**Fig. 4:**
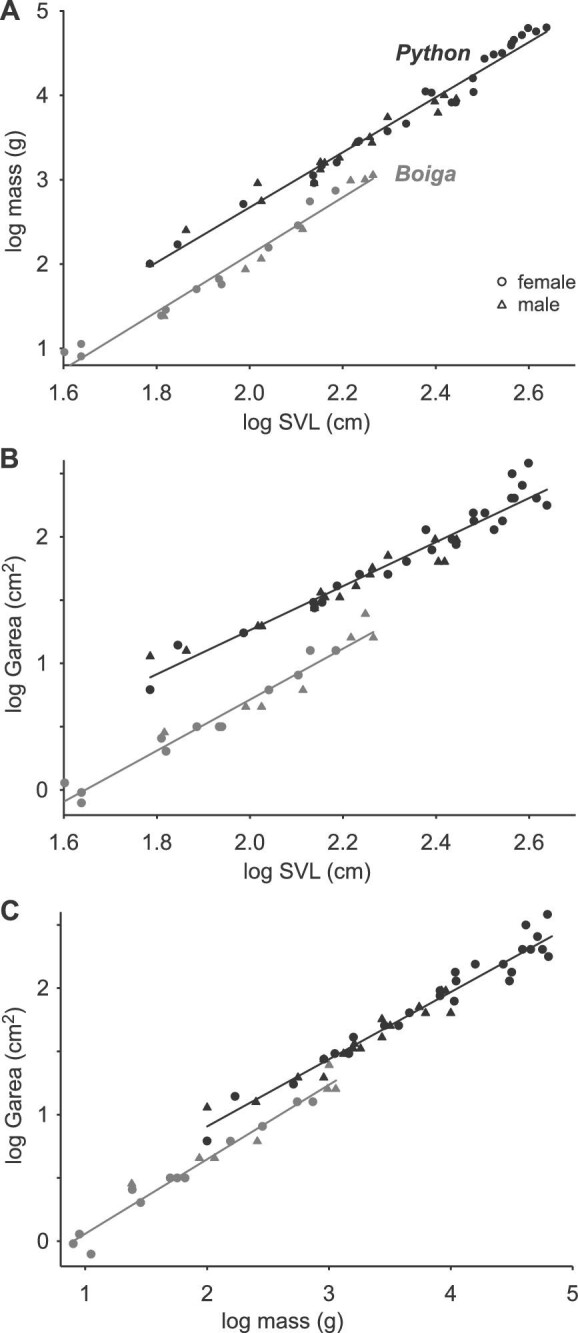
Scaling relationships between overall size and maximal Garea for Burmese pythons (black) and brown treesnakes (gray). (**A**) Mass versus SVL. (**B**) Maximal gape area versus SVL. (**C**) Maximal gape area versus mass. For a given SVL or mass, Burmese pythons had greater mass and Garea than brown treesnakes, but the magnitude of that interspecific difference was less for a given mass than a given SVL. See Table 1 for regression statistics and Table S2 for ANCOVA results.

Table 1 summarizes the scaling relationships of the morphological data for the snakes. For both species, mass scaled with positive allometry relative to SVL (slope > 3). The slope of the regression for brown treesnakes did not differ significantly from that of pythons, and when corrected for SVL, the pythons were significantly heavier than brown treesnakes (Table S2; Fig. 4A). Values of Gdiam of the pythons and brown treesnakes ranged from 2.8–22 cm and 0.9–5.7 cm, respectively (Fig. 1 except the smallest brown treesnake not shown). Relative to SVL, Gdiam and Garea of brown treesnakes scaled isometrically, whereas the values of pythons had negative allometry with slopes that were significantly less than those of brown treesnakes (Table S2; Fig. 4B). Garea of both snake species had negative allometry with mass (Fig. 4C) without the slopes differing significantly between the two species, and the pythons had significantly greater Garea after correcting for mass (Table S2; Fig. 4C). The disparity in Garea between species was greater for a given SVL (Fig. 4B) than for a given mass (Fig. 4C). The residual values of log Gdiam predicted from log SVL revealed that gape after correcting for SVL did not differ significantly between males and females either for Burmese pythons (t = 0.988, df = 41, *P* = 0.329) or for brown treesnakes (t = 0.035, df = 17, *P* = 0.972), and all subsequent analyses used the combined data for males and females. We note, however, that because of sexual size dimorphism in both species, the largest values of gape in each species were attained in the sex with greater overall size (Fig. 4).

**Fig. 5:**
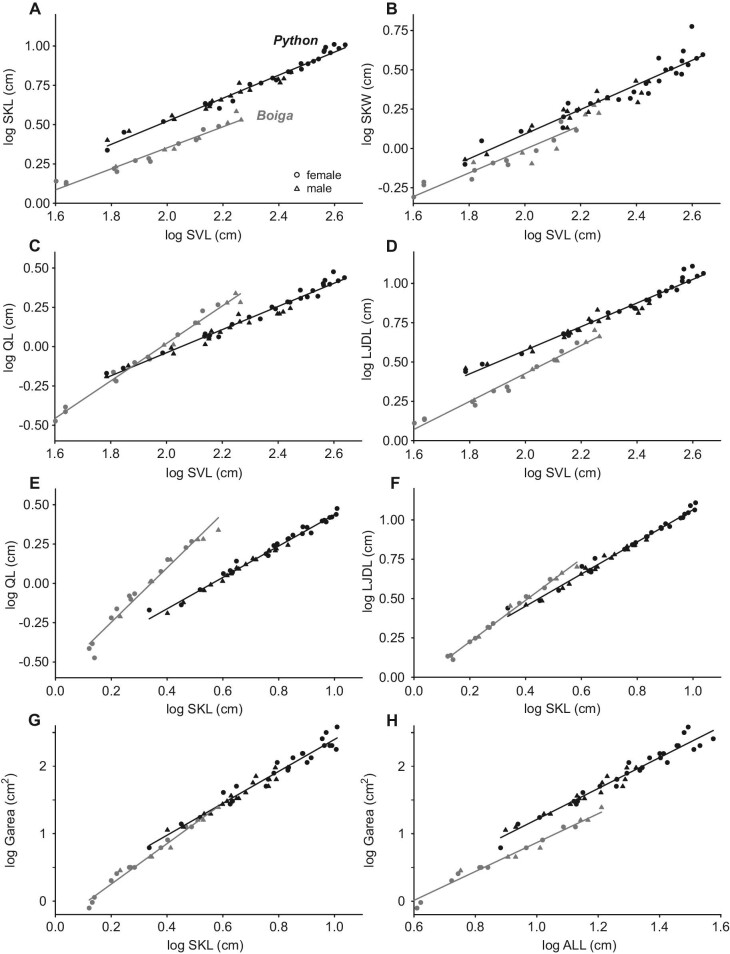
Scaling relationships between measurements of straight-line distances measured at maximal gape. (**A**) Skull length (SKL) versus SVL. (**B**) Skull width (SKW) versus SVL. (**C**) Quadrate length (QL) versus SVL. (**D**) Length of the jaw including the dentary (LJDL) versus SVL. (**E**) Quadrate length (QL) versus skull length (SKL). The slope of the regression for brown treesnakes was significantly steeper than that of Burmese pythons. (**F**) Length of the jaw including the dentary (LJDL) versus skull length (SKL). Maximal Garea versus skull length (**G**) and the combined lengths (ALL) of the left and right jaws and quadrates plus skull width (**H**). For a given SVL the Burmese pythons had significantly longer and wider skulls and longer lower jaws than brown treesnakes. However, even after allowing for the dimensions of the bones contributing to gape, the Burmese pythons still had substantially larger gape than the brown treesnakes (**H**). See Table 1 for regression statistics and Table S2 for ANCOVA results.

Skull length, skull width, and length of the entire lower jaw of both species scaled with negative allometry for SVL (Table 1). For a given SVL, the pythons had significantly longer (Fig. 5A) and wider (Fig. 5B) skulls and longer lower jaws (Fig. 5D) than the brown treesnakes (Table S2). The quadrate length of pythons also scaled with negative allometry relative to SVL, but that of the brown treesnakes had significant positive allometry with a significantly greater slope (Fig. 5C; Table S2). Hence, for values of SVL in common to both species, brown treesnakes had progressively longer quadrates than pythons as SVL increased (Fig. 5C).

The lengths of both the quadrate and entire lower jaw scaled isometrically with skull length (SKL) for the pythons, but brown treesnakes had positive allometry (Table 1) with significantly greater slopes (Table S2). Compared to the pythons with a given SKL, the brown treesnakes had substantially greater values of quadrate length without any overlap with the values for pythons (Fig. 5E), whereas only some of the larger brown treesnakes had slightly longer lower jaws than pythons with similar skull length (Fig. 5F). Compared to brown treesnakes, Garea of pythons at a given SKL was similar (Fig. 5G), but for a given combined length of bones contributing to gape, pythons had significantly larger Garea (Fig. 5H; Table S2).

### How maximal gape is attained

**Fig. 6:**
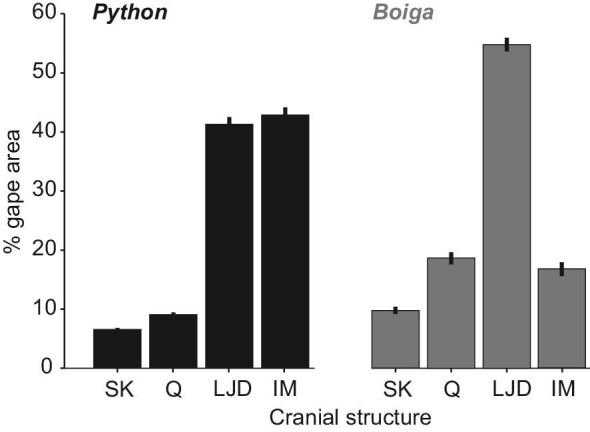
Mean values (±SE) of the relative contributions to maximal Garea of major cranial structures. SK, Q, LJD, and IM indicate the contributions of skull width, the quadrate bone, the entire lower jaw and the intermandibular skin and soft tissues, respectively (Fig. 3A). Values of IM of the Burmese pythons were more than twice those of brown treesnakes, whereas all remaining structures of the brown treesnakes accounted for greater fractions of Garea than the homologous structures of Burmese pythons.

**Fig. 7:**
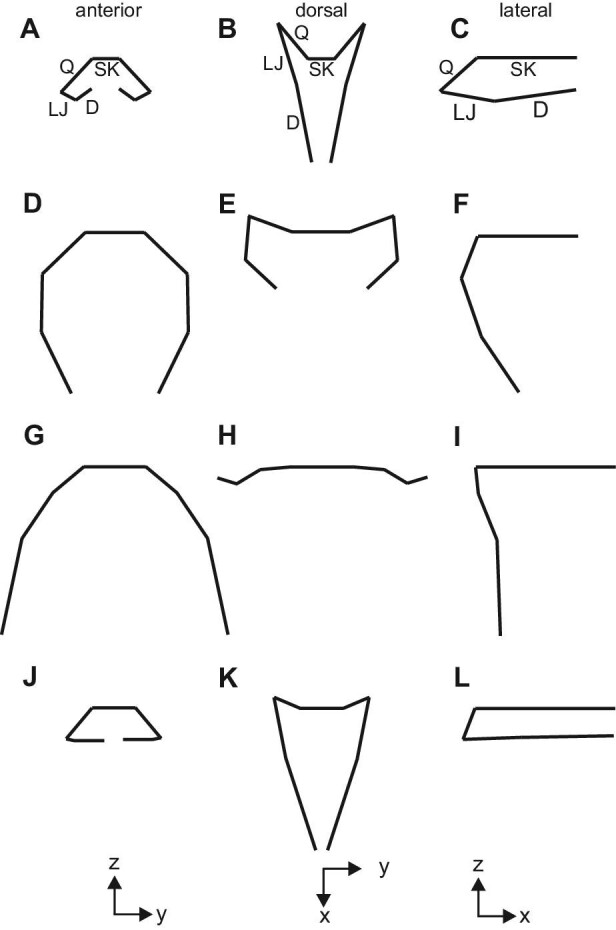
Mean values of angles of major cranial structures at maximal gape and at resting posture as viewed in the transverse, frontal, and sagittal planes. The first and second rows show brown treesnakes at rest (**A**–**C**) and maximal gape (**D**–**F**), respectively, and the third and fourth rows show Burmese pythons at maximal gape (**G**–**I**) and at rest (**J**–**L**), respectively. The dimensions of the figures were standardized for the two species so that the sum of the three-dimensional straight-line lengths of the quadrate (Q), proximal lower jaw (LJ), and dentary (D) were a constant. Within each row images were aligned vertically by the location of the proximal end of the quadrate, and this landmark was also used to align the lateral view images horizontally among all rows. Compared to the brown treesnakes at maximal gape, the Q, LJ, and D of Burmese pythons were more nearly confined to a transverse plane, and the distal ends of these structure were located more laterally relative to their proximal ends. See Table 2 for statistical comparisons between species.

Our findings emphasize the importance of accounting for the inter-mandibular soft tissues (IM) for quantifying maximal gape as the IM mean (and maximum) contributions to Garea were 42.9 ± 0.8% (56%) and 16.8 ± 0.6% (24%) in Burmese pythons and brown treesnakes, respectively (Figs. 1, 6, 7, S3), and these were highly significant differences between the two species (2-tailed t = 23.7, df = 60, *P* < 0001). Values of IM increased with increased SVL, in both Burmese pythons (IM = 0.036*SVL + 34.8, *R*^2^ = 0.475, *n* = 43, *P *< 10**^−^**^6^) and brown treesnakes (IM = 0.032*SVL + 13.5, *R*^2^ = 0.320, *n* = 19, *P* = 0.01). The skin posterior to the head was commonly taut before the skin between the lower jaws. In both species, skull width accounted for less than 10% of Garea (Fig. 6). The lower jaws contributed 41.4 ± 0.7% and 54.7 ± 0.6% in Burmese pythons and brown treesnakes, respectively.

Stretching of the intermandibular soft tissues (Fig. 1, S1) allowed considerable lateral movement of the lower jaws, especially in the Burmese pythons where the distal tips of the dentary and compound bones were lateral to their proximal ends (Fig. 7G). In Burmese pythons at maximal gape, the quadrate and lower jaw bones also appeared nearly vertical in the lateral view (Fig. 7I; Table 2). By contrast, in brown treesnakes at maximal gape (1) the distal tip of the lower jaw was medial and anterior to its proximal end (Fig. 7D, E), (2) the proximal part of the lower jaw was nearly in a parasagittal plane (Fig. 7D), and (3) the distal end of the quadrate was distinctly posterior to its proximal end (Fig. 7F; Table 2).

### Scaling relationships of prey size relative to snake and gape size

**Fig. 8:**
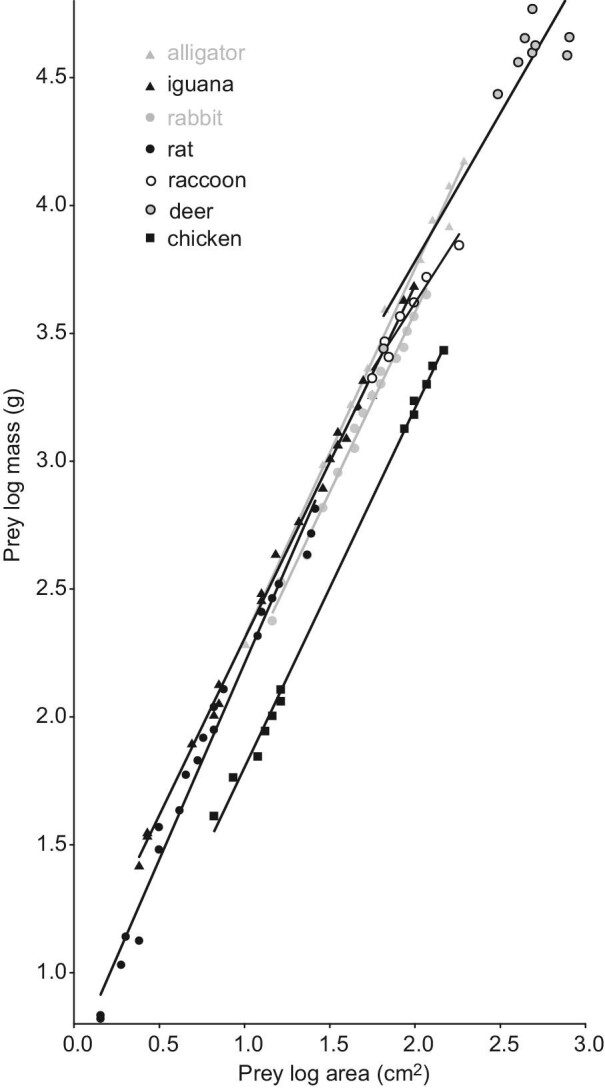
Scaling of mass versus cross-sectional area of potential vertebrate prey items for snakes. For a given cross-sectional area, chickens have a much smaller mass than that of the other taxa. See Table 3 for regressions and Table S3 for ANCOVAs comparing species.

For the five species of potential prey (rat, rabbit, chicken, iguana, and alligator) for which we were able to quantify scaling relationships over a large and nearly continuous range of size, the slopes of mass versus area were quite similar to each other (Table S3). The 95% CL of the slope for rats and alligators included the value of 1.5 expected based on isometry, and the 95% CL of rabbits and chickens were within 0.05 of including 1.5 (Fig. 8; Table 3). Hence, much of the interspecific variation was explained by different elevations of the scaling regressions (Table S3). For example, for a prey diameter = 5 cm (area = 19.6 cm^2^) in common to an alligator, iguana, rat, rabbit, and chicken, the predicted values of mass were 555, 527, 468, 396, and 168 g, respectively. For a diameter = 10 cm (area = 78.5 cm^2^) in common to an alligator, iguana, raccoon, rabbit, chicken, and deer, the predicted masses were 4.012, 3.492, 3.227, 2.454, 1.156, and 4.543 kg, respectively. Thus, for a given prey cross-sectional area, the studied reptiles (iguana and alligator) commonly had the greatest masses, chickens the least, and rats and rabbits were usually intermediate (Table S3). However, deer had greater predicted masses than both raccoons and rabbits with similar area (Fig. 8).

**Table 3:** Ranges of prey size and associated least squares regression statistics (±95%CL) for the scaling equations of log-transformed values of prey mass (g) as a function of prey cross-sectional area (cm*^2^*).

Prey species	*n*	mass (g)	diam (cm)	area (cm^2^)	slope	intercept	*R* ^2^
rat	21	6.6–650	1.4–5.8	1.4–26.0	1.534 ± 0.093	0.675 ± 0.081	0.984
rabbit	17	237–4472	4.2–11.7	14–117	1.405 ± 0.072	0.772 ± 0.124	0.991
raccoon	8	2110–6980	8.5–15.2	56–181	1.039 ± 0.227	1.542 ± 0.447	0.954
chicken	13	41–2720	2.8–12.7	6.6–147	1.404 ± 0.048	0.398 ± 0.077	0.997
iguana	21	26–4810	1.8–11.2	2.4–98.5	1.381 ± 0.039	0.926 ± 0.052	0.996
alligator	11	190–26,600	3.6–19.2	10–289	1.445 ± 0.097	0.865 ± 0.188	0.996
deer*	9	2750–58,600	10.9–35.6 (H) 7.6–29.9 (W)	65–804	1.156 ± 0.397	1.469 ± 1.040	0.872

All *P*-values for the test of the overall significance of the regression were less than 10^–3^. *Area calculated for an ellipse using height (H) and width (W) as the major and minor axes. See Table S3 for ANCOVA results comparing species.

**Fig. 9:**
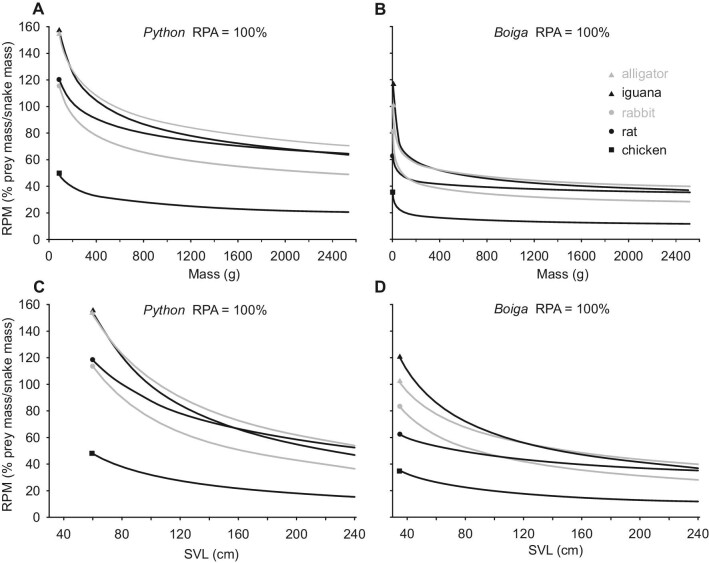
Maximal RPM predicted when relative prey areas equal maximal Garea (RPA = 100%). For both study species and all prey types, scaling relationships (Tables 1, 3) predicted a rapid non-linear decrease in RPM with increased overall snake size. For a given prey type with RPA = 100%, values of RPM for Burmese pythons always exceeded those of brown treesnakes for either a given snake length or snake mass.

**Fig. 10:**
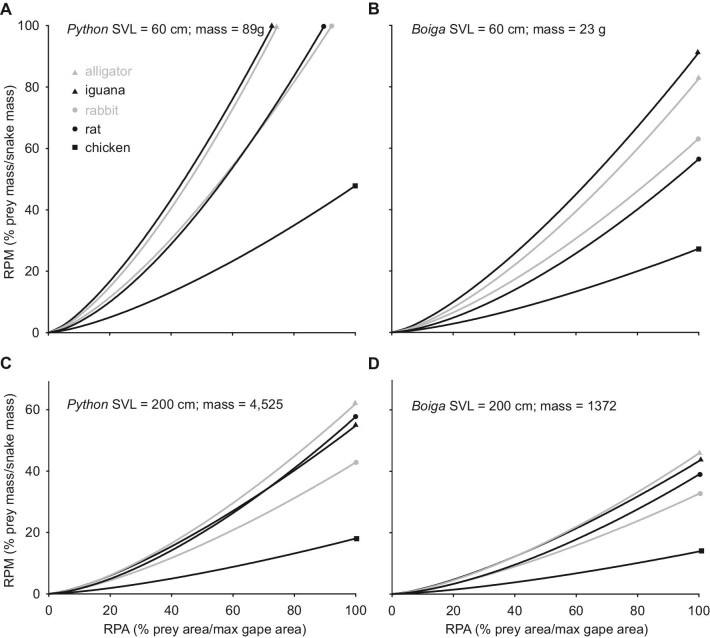
The predicted effects of prey size relative to gape (RPA) on mass of the prey relative to that of the snake (RPM). For both study species and all prey types, scaling relationships (Tables 1, 3) predicted a curvilinear increase in RPM with increased RPA. Hence, the benefit for RPM from a given incremental increase in RPA was greater for a larger compared to a smaller value of RPA, and decreased snake size (panels **C** versus **A** and **D** versus **B**) exaggerated these differences as well as the differences among different prey types. In panel **A**, the values not shown for RPM when RPA = 100% were 154, 152, 117, and 112% for iguanas, alligators, rats, and chickens, respectively. Some biologically unrealistic values of prey size (e.g., in panel **B**, alligators and rabbits are rarely < 40 g, and in panel **C** rats are rarely > 1 kg), were included only to demonstrate the effects of different prey shapes. See Table S4 for additional details.

The following trends occurred for data integrating the scaling relationships of gape, snake size, and prey size (Figs. 9, 10). For both study species and for all prey types when prey cross-sectional area equaled Garea (RPA = 100%), RPM decreased curvilinearly with both increased snake length and mass (Fig. 9). Compared to brown treesnakes with equal mass or SVL, the Burmese pythons always had greater predicted values of RPM (Fig. 9). For both study species and all prey types, as RPA increased, the values of RPM increased curvilinearly (Fig. 10). Decreased snake size within each of the two study species increased the differences among predicted values of RPM for different prey types at a given value of RPA (Fig. 10 panel A vs. C and B vs. D). Compared to brown treesnakes with equal SVL and a given prey type and RPA, pythons always had greater predicted values of RPM (Fig. 10A vs. B; C vs. D).

## Discussion

### Morphological correlates of gape

We deliberately chose two study species with substantial differences in size and shape because two of the most conspicuous sources of morphological variation among snakes are overall size and stoutness (Boback and Guyer 2003; Feldman and Meiri 2013). For example, Burmese pythons were commonly 3–4 times heavier than brown treesnakes with similar SVL. Burmese pythons are rather stout for a python (Table 1, mass = 853 g predicted for SVL = 120 cm) (Esquerre et al. 2017). However, some other pythonid species such as blood pythons (SVL = 120 cm; mass = 3000 g) are even stouter (Shine et al. 1998), and many vipers, especially those in the genus *Bitis*, are also very stout (Bonnet et al. 2001; Feldman and Meiri 2013). On the other extreme, brown treesnakes are rather slender, as is common for arboreal specialists (Lillywhite and Henderson 1993; Pizzatto et al. 2007), but for similar SVL (74 cm) their mass can be more than three times that of the incredibly slender, arboreal colubrid *Imantodes cenchoa* (dos Santos-Costa and da Costa Prudente 2005). Our findings illustrate how these differences in shape and overall size have profound consequences for the interrelationships between gape, potential prey size, and RPM both within and among species.

Theoretically, for snakes with identical length, head size, and gape, a more slender species could have larger value of RPM solely as a result of its small mass rather than large gape. Alternatively, a stouter species could attain parity in RPM with a more slender species if it had a larger head with an attendant larger gape. However, despite Burmese pythons having much larger mass than similar length brown treesnakes, their maximal gape was so large that their predicted values of RPM exceeded rather than merely matching those of the more slender brown treesnake with equal lengths (Figs. 9, 10). Understanding these differences in gape is enhanced by examining the roles of the dimensions, range of motion, and shape of the relevant bones, and the distensibility of the soft tissues between the lower jaws and in the neck.

Head length is a good metric of “overall” head size, but because it is perpendicular to the plane in which the area of the prey must be accommodated, in a strict sense it is irrelevant to gape. Nonetheless, a common correlate of a longer head is a longer lower jaw, which has great relevance to gape (Fig. 7). Additional mechanisms for accommodating a longer lower jaw include lengthening the supratemporal and lengthening the quadrate and orienting it so that the distal end is increasingly posterior to the proximal end. Although the Burmese pythons had larger gape, brown treesnakes had both longer quadrate bones (Figs. 2, 5) and a more posteriorly oriented distal end of the quadrate in the resting position (Fig. 7). Head length is one of many linear cranial measurements that have been used individually or in combination as surrogates for the gape of snakes (King 2002; Vincent et al. 2006; Barends and Maritz 2021). However, it may be most useful to view increased head length as only having an indirect effect on gape by accommodating a longer lower jaw. By contrast, skull width, quadrate length, and lower jaw length are all directly relevant to maximal gape (Fig. 7), but the maximal gape of Burmese pythons for a given combined length of these structures was still much greater than that of brown treesnakes (Fig. 5H). Consequently, even these skeletal dimensions that clearly contribute to maximal gape are not sufficient to explain interspecific variation in gape.

Although a detailed study of the range of motion of the bones contributing to gape is beyond the scope of this study, when we manipulated the bones of freshly euthanized specimens, we gained the following insights. In both of our study species, the distal ends of the supratemporal bones could move laterally so that the width of the skull was as much as 30% greater at maximal gape compared to the resting position (Fig 7D vs. A; G vs. J). A common presumption is that significant mobility of the snake supratemporal bone is absent except in some highly specialized snakes in the genus *Dasypeltis* that are obligate egg eaters (Gans 1974); hence, mobility of the supratemporal merits further study. In both of our study species, the tips of the lower jaws could expand laterally much more when the skin between them was cut rather than intact. This raises the interesting possibility that the skin, rather than the morphology of the joint, limits the range of motion of some bones that contribute to gape.

Both of our study species and most snake species have curved lower jaws, and the limited data available suggest this curvature is quite similar to that of the cylindrical mouth opening at maximal gape (Jayne et al. 2018; Gripshover and Jayne 2021). Part of the apparent curvature of the lower jaws arises from the mobility of the dentary bone relative to the compound bone, but additional curvature exists within the bones themselves (Figs. 2, 3). With the proper orientation, such curvature can enhance gape significantly as can be illustrated by how a straight line from the proximal to distal end of the entire lower jaw would intrude into the Garea (Fig. 3A).

The importance of the soft tissues for determining gape has been long been recognized (Gans 1974; Arnold 1983; Cundall and Greene 2000; Close and Cundall 2014). However, the near absence of empirical data for both maximal gape and what contributes to it (e.g., Figs. 3A, 6) has impeded clearly understanding the evolution of macrostomy in snakes and the most significant morphological correlates of large gape. Whether the tremendous distension of the soft tissue in Burmese pythons is a characteristic only of the giant species of boids and pythonids or whether this condition is nearly universal in boas and pythons would be interesting to resolve. Furthermore, it would be interesting to determine if values for the contribution of the soft tissues to maximal Garea of diverse snakes form a continuum of variation or have a discontinuity or some threshold value that is consistent with the taxa of snakes designated as macrostomates.

Given the phylogenetic distance and morphological differences between our two study species, finding a difference in gape was not surprising. However, we did not anticipate either the large observed magnitude of the differences in gape or that the soft tissue of the Burmese pythons would contribute more than twice as much to maximal Garea as in the brown treesnake. Such differential importance of the soft tissues for contributing to maximal gape further emphasizes the inadequacy of using only skeletal anatomy to address issues related to variation in gape among different snake species.

### Ecological implications

Clearly gape can impose an anatomical limit on the prey size of snakes, but a single metric of prey size is rarely sufficient to understand size relationships between predators and prey because different prey species often have different shapes. What predators actually consume also depends both on what is available and the behavior of the animals, and both of our study species are generalist predators that consume a wide variety of vertebrate prey with substantial differences in shape. For example, brown treesnakes eat frogs, lizards, birds, bird eggs, bats, and quadrupedal mammals, but as snake size increases the consumption of ectothermic vertebrates decreases as endothermic vertebrates comprise increasingly more of their diet (Savidge 1988; Greene 1989). Burmese pythons eat several species of birds and occasionally bird eggs (Dove et al. 2011), large species such as deer (Boback et al. 2016; Bartoszek et al. 2018) and a wide variety of mammals as well as frogs, lizards, and alligators (Snow et al. 2007). Unfortunately, where these versatile predators have been introduced, brown treesnakes have decimated avian populations in Guam (Savidge 1987), and mammal populations have declined precipitously where Burmese pythons occur in south Florida (Dorcas et al. 2012).

Besides the variable mass of prey within and among species, the shapes of prey species have profound implications for the foraging ecology of gape-limited predators such as snakes. For example, some extant non-macrostomate snake species in the genera *Anilius* and *Cylindrophis* commonly consume elongate prey such as caecilians, amphisbaenians, snakes, and eels (Greene 1983; Maschio et al. 2010; Hampton 2018b). Highly specialized sea snakes with very small heads and narrow necks mostly eat elongate species of eels (Voris and Voris 1983; Sherratt et al. 2019). Thus, for certain species of snakes or for small snakes within a species that have small gape, consuming elongate prey provides a proximate mechanism for increasing RPM for a given gape. Available data suggest that lizards are often more elongate (greater mass per cross-sectional area) than many birds and mammals (Fig. 8). Hence, the greater proportion of lizards in the diet of small compared to large brown treesnakes (Savidge 1988; Greene 1989) is consistent with this strategy, and similar ontogenetic shifts in diet from ectothermic to endothermic vertebrates are quite common in diverse species of snakes (Klauber 1972; Broadley 1983; Shine 1991).

Evolving larger gape for a given snake mass provides another (ultimate) mechanism for increasing RPM, and increased RPM can decrease the frequency of feeding (Greene 1983). Several previous studies of RPM have also commonly discussed the benefits of increased gape for facilitating the consumption of “bulky” prey. However, bulky prey has variously referred to (1) prey that are large relative to the gape of the snake, (2) prey that have large mass relative to that of the snake, (3) prey with a large disparity in height versus width, and (4) prey that are difficult to swallow (Cundall and Greene 2000; Mori and Vincent 2008; Willson and Hopkins 2011). Rather than using the term bulky for all these different attributes of prey that can be decoupled from each other, we favor specifically using RPA, RPM, cross-sectional aspect ratio, and irregular shape (such as protruding appendages that complicate swallowing), respectively. Additional features, such as how elongate or stout prey are, can be described by relating mass to cross-sectional area. Given the continuous variation in so many attributes of prey that are relevant to foraging ecology, rather than using prey attributes to define discrete types or categories of prey, it could be instructive to examine the continuum of variation that they provide within and among species.

Even our very limited sample of potential prey species had substantial differences in the relationship of mass to cross-sectional area. Of the species that we measured, presumably the large muscular tails of alligators and iguanas contributed to their large mass for a given cross-sectional area compared to the endothermic species that we measured, and chickens had by far the lowest mass for a given cross-sectional area (Fig. 8). Hence, one might expect that snakes specializing on birds would have relatively large gape to enhance RPM for such stout prey. However, many arboreal snakes that consume birds, such as brown treesnakes and Amazon tree boas (*Corallus hortulanus*), have convergently evolved bodies that are light for their length (Lillywhite and Henderson 1993; Pizzatto et al. 2007). Hence, prey with a modest mass per cross-sectional area could still have reasonably large values of RPM for slender arboreal species compared to stouter species of snakes with similar gape, but we still lack the empirical data on gape with attendant methods accounting for phylogeny that are needed to test this rigorously.

To attain a particular value of RPM, trophically specialized and generalist snakes can exploit ontogenetic variation in size that occurs within a single prey species. However, trophic generalists such as our study species also have a wide variety of potential prey that affords many additional options for attaining a particular value of RPM for a given snake size or as the snakes grow larger. The following examples provide some estimates of the limits on different size prey and how the combination of variation in gape, prey type, and prey size affect RPM.

Some of the inter-individual variability in gape and size (Table S1) resulted in values departing noticeably from the curves predicting RPM for different prey types, RPA, and snake size based solely on the scaling equations (Fig. 9). For example, two pythons with Gdiam = 5 cm and similar SVL (104 and 106 cm) had large differences in mass (908 and 556 g); hence, for the same gape and when RPA = 100%, disparate values of RPM (97 and 60%) were predicted for consuming an alligator (Table S1). Other pythons with similar SVL (365 and 366 cm) and reasonably similar masses (38.8 and 41.3 kg) had substantially different Gdiam (16 and 20 cm) with correspondingly large differences in maximal predicted RPM (Table S1). The Burmese python with the largest observed value of Gdiam (22 cm) had SVL = 397 cm, mass = 63.3 kg, and a maximal predicted RPM of 63% for a 39 kg alligator (Table S1). Overall, the scaling data alone suggest that Burmese pythons with SVL > 150 cm would be unlikely to eat prey with RPM > 100% (Table S4).

From similar scaling data, we gained further insights into what prey brown treesnakes should be able to swallow (Table S4). For example, the predicted Garea of brown treesnakes does not equal that of a hatchling chicken (mass ca 35 g) until SVL exceeds 100 cm. However, a snake near neonatal size (SVL < 40 cm) has predicted Garea that is similar to that a 6 g neonatal rat and a 10 g lizard the size and shape of a neonatal iguana. Our largest observed Gdiam was 5.7 cm while SVL = 177 cm and mass = 1003 g, and the corresponding Garea matched those of a 739 g iguana (RPM = 74%), an enormous rat, (1000 g; RPM = 100%) and a 236 g chicken (RPM = 24%). In the lab of BCJ a long-term captive brown treesnake (directly measured Gdiam = 5 cm, SVL = 173 cm, mass = 960 g) tried three times to swallow a 330 g 4.5-cm diameter rat (RPA = 90%) headfirst, but it failed, probably from the combination of a localized bulge in the rat abdomen (Close and Cundall 2012) and an inability to move the jaws over bumps created by the bases of the hind limbs (Fig. S1). Unfortunately, for brown treesnakes we lack sufficiently detailed field observations to provide further insights into maximal prey size (RPA) actually consumed by the snakes.

Our scaling data emphasize that smaller snakes gain larger benefits for RPM from a given relative increase in maximal gape (Fig. 10; Table S4). For example, increasing Gdiam 10% for Burmese pythons predicts that RPM of rats increases from 134 to 183% for a hatchling (SVL = 60 cm, mass = 100 g, Gdiam = 3.1 cm) and RPM of rabbits increases from 42 to 57% for a larger snake (SVL = 200 cm, mass = 4,500, Gdiam = 8.8 cm). A 10% increase in Gdiam of brown treesnakes predicts RPM of iguanas increases from 104–135% for a small individual (SVL = 50 cm, mass = 12 g, Gdiam = 1.3 cm) and from 50 to 65% for a larger individual (SVL = 150 cm, mass = 520 g, Gdiam = 3.9 cm).

Further insights regarding maximal prey size come from the following field observations of Burmese pythons in southern Florida. Case A provides an interesting example of a modest size snake exploiting a small individual of a species with large maximal size. One of us (IB) found a python (SVL = 189 cm, mass = 4.5 kg, estimated Garea = 55.5 cm^2^) that had recently eaten a white-tailed deer with observed (obs) values of cross-sectional area = 65 cm^2^, mass = 2.65 kg, and RPM = 59% and an estimated (est) value of RPA = 117%. In case B (Bartoszek et al. 2018) a snake (obs SVL = 294 cm, obs mass = 14.3 kg, est Garea = 120 cm^2^) regurgitated a recently consumed deer fawn (obs mass = 15.9 kg, est area = 231 cm^2^), for which obs RPM = 111% and est RPA = 231%. Case C involves a widely publicized photo (Snow et al. 2007) of a dead and ruptured snake (obs total length = 396; est SVL = 342 cm, est mass = 25.8 kg, est Garea = 156 cm^2^) containing an alligator (obs total length = 196 cm, est area = 252 cm^2^, est mass = 22 kg) with estimated values of RPA = 161% and RPM = 85%.

For cases A, B and C the upper 95% CL of predicted Garea from the scaling regression with SVL were 60,131, and 173 cm^2^, respectively, and this nearly explained the feasibility of case A but was not sufficient to explain the other cases. However, another important source of inter-individual variation is that IM varied considerably (29–56%) even for some snakes with very similar SVL. Hence, to better understand the importance of IM as a determinant of maximal gape, for all pythons preserved at maximal gape, we determined the arc length of all structures excluding the intermandibular soft tissues and used this length as 44% of the circumference of a circle that was subsequently constructed and used to recalculate the scaling relationship of Garea when IM = 56% (log Garea = 1.497*log SVL 1 1.437, *R*^2^ = 0.965, *P* << 0.0001). For cases A, B, and C, these adjusted estimates of Garea assuming IM = 56% were 94,182 and 228 cm^2^, respectively, and these adjusted values may readily explain case A and nearly explain the feasibility of case C. However, assuming the skeletal dimensions of the snake in case B are similar to the snakes in our sample, then we estimate that a value of IM just over 60% would be required to swallow this deer with an extraordinarily large RPM.

Our largest observed Gdiam was 22 cm for a python with SVL = 397 cm, but for this specimen IM was only 51%. If IM of this snake were increased to 56%, then estimated Gdiam = 24.3 cm, and the resulting value of Garea could allow swallowing a 36 kg deer or a 52 kg alligator. The non-IM arc length for the snake with SVL = 397 cm was 33.6 cm and that of another snake (SVL = 370 cm) was 30.1 cm. If this incremental change in non-IM arc length with change in SVL combined with IM = 50% were used to extrapolate values for the longest Burmese python caught in Florida (total length = 576, est SVL = 495 cm), the resulting estimate of Gdiam = 34.6 cm could theoretically permit swallowing an 80 kg deer.

These collective observations of gape size and prey size clearly demonstrate that at least some individual Burmese pythons do indeed occasionally consume impressively large prey that tax their anatomical limit for prey size. However, a key unresolved issue is how often such events occur. Although snakes have large maximal gape, in nature snakes may or may not choose prey sizes that tax this maximal capacity. This general issue of the extent to which animals function near a maximal capacity in nature (Hertz et al. 1988) has rarely been tested for any functional system including the feeding of snakes. For the foraging ecology of snakes, a promising method for addressing this general issue of performance in nature is quantifying the frequency distribution of RPA for consumed prey. However, to date this has only been done for four species of very specialized species of crustacean-eating snakes, all of which consume prey with shapes that preclude attaining values of RPM > 70% even when RPA = 100% for the smallest individuals of each of these species (Gripshover and Jayne 2021). In the field, the two species that eat freshly molted crustaceans often had prey near maximal values of RPA, whereas the two species that eat hard-shelled crustaceans always ate sizes of prey in the field well below the maximal size predicted based on maximal gape (Jayne et al. 2018; Gripshover and Jayne 2021). Such a complete absence of prey with RPA > 70% (Gripshover and Jayne 2021) might be explained by an inability of the snakes to successfully capture and subdue larger, more formidable and more mobile prey rather than being limited by their anatomy. By contrast, a significant number of precedents exist for pythons in the wild attacking or killing and attempting to eat prey larger than can be swallowed (Natusch et al. 2021). Thus, generalities regarding the foraging ecology of snakes after rigorously accounting for maximal gape and phylogeny largely remain to be determined. However, the phylogenetic diversity of snakes, and the wide variety of snake morphology and prey consumed still hold great promise as a model system for testing the role of anatomical constraints on prey size and foraging tactics.

## Supplementary Material

obac033_Supplemental_Files

## References

[bib1] Arnold SJ. 1983. Morphology, performance, and fitness. Am Zool 23:347–61.

[bib2] Barends JM , Maritz B. 2021. Specialized morphology, not relatively large head size, facilitates competition between a small-bodied specialist and large-bodied generalist competitors. J Zool 315:213–24.

[bib3] Barker DG , Barten SL, Ehrsam JP, Daddono L. 2012. The corrected lengths of two well-known giant pythons and the establishment of a new maximum length record for Burmese pythons, *Python bivittatus*. Bull Chicago Herp Soc 47:1–6.

[bib4] Bartoszek IA , Andreadis PT, Prokopervin C, Patel M, Reed RN. 2018. *Python bivittatus* (Burmese python). Diet and prey size. Herp Rev 49:139–40.

[bib5] Boback SM , Guyer C. 2003. Empirical evidence for an optimal body size in snakes. Evol 57:345–451. 10.1111/j.0014-3820.2003.tb00268.x 12683530

[bib6] Boback SM , Snow RW, Hsu T, Peurach SC, Dove CJ, Reed RN. 2016. Supersize me: remains of three white-tailed deer (*Odocoileus virginianus*) in an invasive Burmese python (*Python molurus bivittatus*) in Florida. Bioinvasions Rec 5:197–203.

[bib7] Bonner JT. 2006. Why Size Matters: From Bacteria to Blue Whales Princeton, NJ: Princeton University Press.

[bib8] Bonnet X , Shine R, Naulleau G, Thiburce C. 2001. Plastic vipers: influence of food intake on the size and shape of Gaboon vipers (*Bitis gabonica*). J Zool 255:341–51.

[bib9] Broadley DG. 1983. Fitzsimmon's Snakes of Southern Africa. Johannesburg: Delta Books (PTY) LTD.

[bib10] Calder WA. 1984. Size, Function, and Life History. Cambridge, MA: Harvard University Press.

[bib11] Caldwell MW. 2020. The origin of snakes morphology and the fossil record. Boca Raton CRC Press, Taylor and Francis Group.

[bib12] Close M , Cundall D. 2012. Mammals as prey: estimating ingestible size. J Morphol 273:1042–9. 22729897 10.1002/jmor.20042

[bib13] Close M , Cundall D. 2014. Snake lower jaw skin: extension and recovery of a hyperextensible keratinized integument. J Exp Zool A 321:78–97. 10.1002/jez.1839 24497479

[bib14] Colston TJ , Costa GC, Vitt LJ. 2010. Snake diets and the deep history hypothesis. Biol J Linn Soc 101:476–86.

[bib15] Cundall D. 2019. A few puzzles in the evolution of feeding mechanisms in snakes. Herpetologica 75:99–107.

[bib16] Cundall D , Greene HW. 2000. Feeding in snakes. In: Schwenk K, editor. Feeding Form, Function, and Evolution in Tetrapod Vertebrates. New York: Academic Press. p. 293–333.

[bib17] Dorcas ME , Willson JD, Reed RN, Snow RW, Rochford MR, Miller MA, Meshaka WE, Andreadis PT, Mazzotti FJ, Romagosa CM et al. 2012. Severe mammal declines coincide with proliferation of invasive Burmese pythons in Everglades National Park. Proc Natl Acad Sci USA 109:2418–22. 22308381 10.1073/pnas.1115226109 PMC3289325

[bib18] dos Santos-Costa MC , da Costa Prudente AL. 2005. *Imantodes cenchoa* (Chunk-headed snake) mating. Herp Rev 36:324.

[bib19] Dove CJ , Snow RW, Rochford MR, Mazzotti FJ. 2011. Birds consumed by the invasive Burmese python (*Python molurus bivittatus*) in Everglades National Park, Florida, USA. Wilson J Ornithol 123:126–31.

[bib20] Esquerre D , Sherratt E, Keogh JS. 2017. Evolution of extreme ontogenetic allometric diversity and heterochrony in pythons, a clade of giant and dwarf snakes. Evol 71:2829–44. 10.1111/evo.13382 29076160

[bib21] Feldman A , Meiri S. 2013. Length-mass allometry in snakes. Biol J Linn Soc 108:161–72.

[bib22] Gans C. 1974. Biomechanics. An Approach to Vertebrate Biology. Ann Arbor: University of Michigan Press.

[bib23] Greene HW. 1983. Dietary correlates of the origin and radiation of snakes. Am Zool 23:431–41.

[bib24] Greene HW. 1989. Ecological, evolutionary, and conservation implications of feeding biology in old world cat snakes, genus *Boiga* (Colubridae). Proc Calif Acad Sci 46:193–207.

[bib25] Gripshover ND , Jayne BC. 2021. Crayfish eating in snakes: testing how anatomy and behavior affect prey size and feeding performance. Integr Org Biol 3:1–16. 10.1093/iob/obab001 PMC8023418 33842838

[bib26] Grundler MC , Rabosky DL. 2021. Rapid increase in snake dietary diversity and complexity following the end-Cretaceous mass extinction. PLoS Biol 19:e3001414. 34648487 10.1371/journal.pbio.3001414 PMC8516226

[bib27] Hampton P. 2018a. Morphological indicators of gape size for red-tailed pipe snakes (*Cylindrophis ruffus*). J Herpetol 52:425–9.

[bib29] Hampton PM. 2018b. Morphological indicators of gape size for red-tailed pipe snakes (*Cylindrophis ruffus*). J Herpetol 52:425–9.

[bib28] Hampton P , Kalmus T. 2014. The allometry of cranial morphology and gape size in red-bellied mudsnakes (*Farancia abacura*). Herpetologica 70:290–7.

[bib30] Hampton PM , Moon BR. 2013. Gape size, its morphological basis, and the validity of gape indices in western diamond-backed rattlesnakes (*Crotalus atrox*). J Morphol 274:194–202. 23108999 10.1002/jmor.20087

[bib31] Headland TN , Greene HW. 2011. Hunter-gatherers and other primates as prey, predators, and competitors of snakes. Proc Natl Acad Sci USA 108:E1470–4. 22160702 10.1073/pnas.1115116108 PMC3248510

[bib32] Hertz PE , Huey RB, Garland T. 1988. Time budgets, thermoregulation, and maximal locomotor performance—are reptiles olympians or boy scouts. Am Zool 28:927–38.

[bib33] Hsiang AY , Field DJ, Webster TH, Behlke ADB, Davis MB, Racicot RA, Gauthier JA. 2015. The origin of snakes: revealing the ecology, behavior, and evolutionary history of early snakes using genomics, phenomics, and the fossil record. BMC Evol Biol 15:10.1186/s12862-015-0358-5. 10.1186/s12862-015-0358-5 PMC4438441 25989795

[bib34] Huxley JS , Teissier G. 1936. Terminology of relative growth. Nature 137:780–1.

[bib35] Jayne BC , Voris HK, Ng PKL. 2018. How big is too big? Using crustacean-eating snakes (*Homalopsidae*) to test how anatomy and behavior affect prey size and feeding performance Biol J Linn Soc 123:636–50.

[bib36] Kilmer JT , Rodriguez RL. 2017. Ordinary least squares regression is indicated for studies of allometry. J Evol Biol 30:4–12. 27711984 10.1111/jeb.12986

[bib37] King RB. 2002. Predicted and observed maximum prey size: snake size allometry. Funct Ecol 16:766–72.

[bib38] Klauber L. 1972. Rattlesnakes their habits, life histories, and influence on mankind. Berkeley: University of California Press.

[bib39] Lillywhite HB , Henderson RW. 1993. Behavioral and functional ecology of arboreal snakes. In: Seigel RA, Collins JT, editors. Snakes - Ecology and Behavior. New York (NY): McGraw Hill Inc. p. 1–48.

[bib40] Maschio GF , Prudente ALD, Rodrigues FD, Hoogmoed MS. 2010. Food habits of *Anilius scytale* (*Serpentes: Aniliidae*) in the Brazilian Amazonia. Zoologia 27:184–90.

[bib41] Mori A , Vincent SE. 2008. An integrative approach to specialization: relationships among feeding morphology, mechanics, behaviour, performance, and diet in two syntopic snakes. J Zool 275:47–56.

[bib42] Natusch D , Lyons J, Mears LA, Shine R. 2021. Biting off more than you can chew: attempted predation on a human by a giant snake (*Simalia amethistina*). Austral Ecol 46:159–62.

[bib43] Pittman SE , Bartoszek IA. 2021. Initial dispersal behavior and survival of non-native juvenile Burmese pythons (*Python bivittatus*) in South Florida. BMC Zool 6:1–13. 10.1186/s40850-021-00098-2 PMC10124209 37170339

[bib44] Pizzatto L , Almeida-Santos SM, Shine R. 2007. Life-history adaptations to arboreality in snakes. Ecol 88:359–66. 10.1890/0012-9658(2007)88[359:latais]2.0.co;2 17479754

[bib45] Rivas Ja . 2020. Anaconda: The Secret Life of the World's Largest Snake. New York (NY): Oxford University Press.

[bib46] Rodda GH , Fritts TH, McCoid MJ, Campbell EWI. 1999a. An overview of the biology of the Brown Treesnake (*Boiga irregularis*), a costly introduced pest on Pacific islands. In: Rodda GH, Sawai Y, Chiszar D, Tanaka H, editors. Problem Snake Management: The Habu and the Brown Treesnake. Ithaca: Cornell University Press. p. 44–80.

[bib47] Rodda GH , Sawai Y, Chiszar D, Tanaka H. 1999b. Problem Snake Management: The Habu and the Brown Treesnake. Ithaca: Cornell University Press.

[bib48] Ryerson WG. 2020. Captivity affects head morphology and allometry in headstarted garter snakes, *Thamnophis sirtalis*. Integr Comp Biol 60:476–86. 32321171 10.1093/icb/icaa020

[bib49] Savidge JA. 1987. Extinction of an island forest avifauna by an introduced snake. Ecology 68:660–8.

[bib50] Savidge JA. 1988. Food habits of *Boiga irregularis*, an introduced predator in Guam. J Herpetol 22:275–82.

[bib51] Schmidt-Nielsen K. 1984. Scaling: Why Is Animal Size so Important? Cambridge: Cambridge University Press.

[bib52] Secor SM. 1995. Digestive response to the first meal in hatchling Burmese pythons (*Python molurus*). Copeia: 1995:947–54.

[bib53] Sherratt E , Sanders KL, Watson A, Hutchinson MN, Lee MSY, Palci A. 2019. Heterochronic shifts mediate ecomorphological convergence in skull shape of microcephalic sea snakes. Integr Comp Biol 59:616–24. 31065670 10.1093/icb/icz033

[bib54] Shine R. 1991. Australian Snakes: A Natural History. Chatswood, NSW: Reed Books.

[bib55] Shine R , Ambariyanto, Harlow PS, Mumpuni. 1998. Ecological divergence among sympatric colour morphs in blood pythons, *Python brongersmai*. Oecologia 116:113–9. 28308515 10.1007/s004420050569

[bib56] Snow RW , Krysko KL, Enge KM, Oberhofer L, Warren-Bradley A, Wilkins L. 2007. Introduced populations of *Boa constrictor* (Boidae) and *Python molurus bivittatus* (Pythonidae) in southern Florida. In: Henderson RW, Powell R, editors. Biology of the Boas and Pythons. Eagle Mountain, UT: Eagle Mountain Publishing, LC. p. 417–38.

[bib57] Taillie PJ , Hart KM, Sovie AR, McCleery RA. 2021. Native mammals lack resilience to invasive generalist predator. Biol Conserv 261:109290.

[bib58] Vincent SE , Dang PD, Herrel A, Kley NJ. 2006. Morphological integration and adaptation in the snake feeding system: a comparative phylogenetic study. J Evol Biol 19:1545–54. 16910984 10.1111/j.1420-9101.2006.01126.x

[bib59] Voris HK , Voris HH. 1983. Feeding strategies in marine snakes: an analysis of evolutionary, morphological, behavioral, and ecological relationships. Am Zool 23:411–25.

[bib60] Wilkinson L. 1992. SYSTAT for windows: Statistics, Version 5 Edition. Evanston, Illinois: SYSTAT Inc.

[bib61] Willson JD , Hopkins WA. 2011. Prey morphology constrains the feeding ecology of an aquatic generalist predator. Ecology 92:744–54. 21608482 10.1890/10-0781.1

[bib62] Zaher H , Murphy RW, Arredondo JC, Graboski R, Machado PR, Mahlow K, Montingellil GG, Quadros AB, Orlov NL, Wilkinson M et al. 2019. Large-scale molecular phylogeny, morphology, divergence-time estimation, and the fossil record of advanced caenophidian snakes (*Squamata: Serpentes*). PLoS One 14:10.1371/journal.pone.0216148. 10.1371/journal.pone.0216148 PMC6512042 31075128

