# Supplementary material for: Scaling Relationships of Maximal Gape in Two Species of Large Invasive Snakes, Brown Treesnakes and Burmese Pythons, and Implications for Maximal Prey Size
Source: Integr Org Biol. 2022 Aug 25;4(1):obac033. doi: 10.1093/iob/obac033 (PMC9409080; doi:10.1093/iob/obac033)
Supplement: obac033_Supplemental_Files [file obac033_supplemental_files.zip › Fig_S1.pdf]

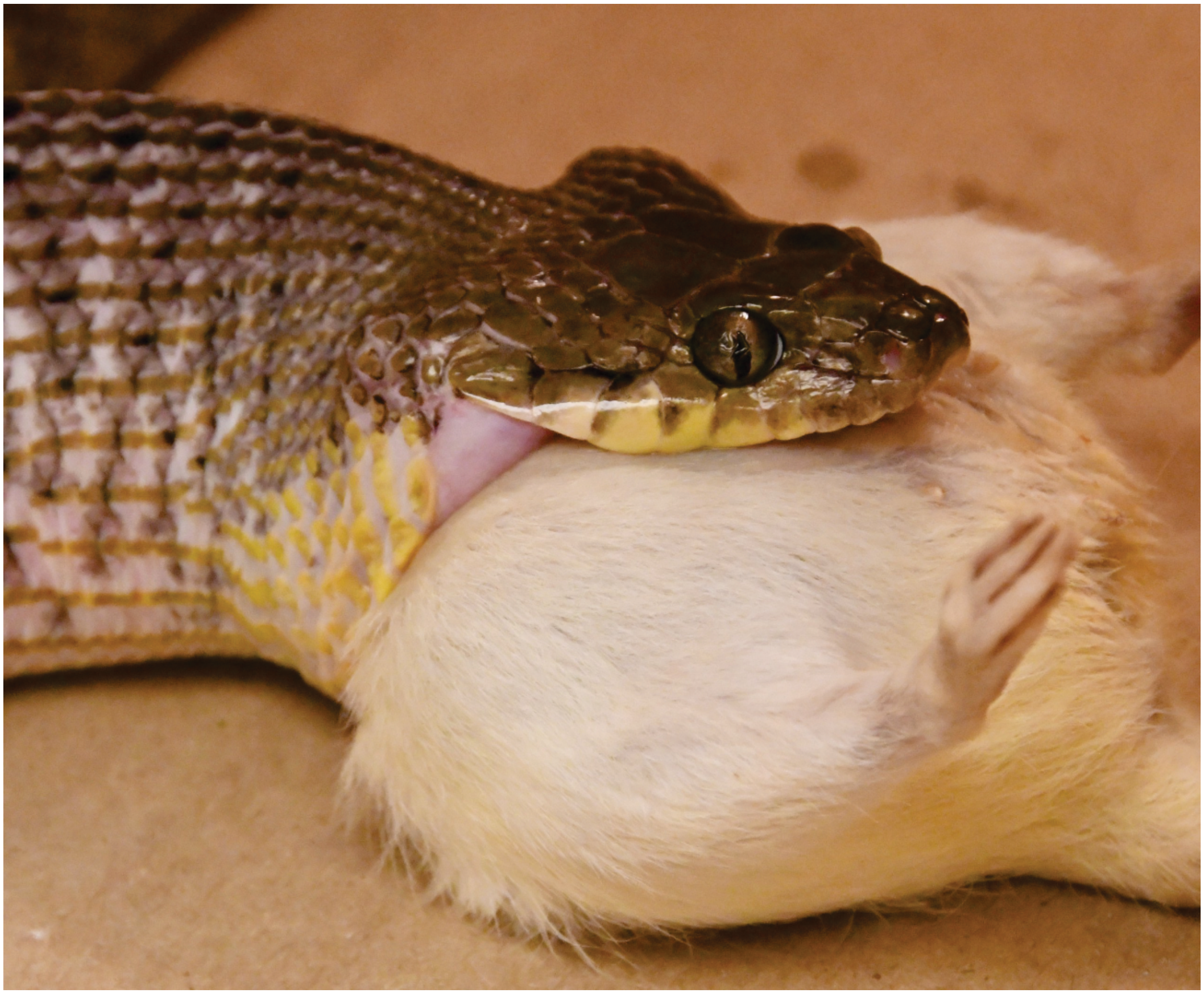

Fig. S1 A dead rat that was too large for a brown treesnake to swallow despite three attempts to do so. The snake was a long-term captive with measured values of SVL = 173 cm, mass = 960 g and Gdiam = 5 cm. The rat had directly measured values of mass = 330 g and circular diameter = 4.5 cm. Note the hips of the rat are at the edge of the lower jaw, and the abdomen is bulging significantly as Close and Cundall (2012) described previously for mammalian prey being swallowed by snakes.
