# Supplementary material for: Scaling Relationships of Maximal Gape in Two Species of Large Invasive Snakes, Brown Treesnakes and Burmese Pythons, and Implications for Maximal Prey Size
Source: Integr Org Biol. 2022 Aug 25;4(1):obac033. doi: 10.1093/iob/obac033 (PMC9409080; doi:10.1093/iob/obac033)
Supplement: obac033_Supplemental_Files [file obac033_supplemental_files.zip › FINAL_IOB-2022-008_legends.docx]

**Figure legends**

**Fig. 1** Range of sizes used to determine maximal gape. The inset shows brown treesnakes (SVL = 40, 177 cm; gape = 1.2, 5.7 cm), whereas the Burmese pythons (SVL = 61, 397 cm; gape = 2.8, 22 cm) are in the background. All images are to the same scale. The arrow indicates the distal end of the lower jaw in the large python. The small python contained substantial yolk. At maximal gape, the circumferential distance between scales exceeded the width of scales in the neck, whereas the scales at rest completely covered the regions of skin in between them.

**Fig. 2** CT scans showing the landmarks (dots) used for morphological measurements. (**A**, **B**) Burmese python (SVL = 61 cm). (**C**,**D**) Brown treesnake (SVL = 130 cm). Both specimens had a maximal gape diameter of 2.8 cm. In the hatchling python, the proximal quadrate did contact the supratemporal bone, but the cartilaginous end of the quadrate is not visible in this rendering. Skull length (SKL) was the distance along the mid-dorsal line from the snout to the dorso-posterior margin of the parietal bones, whereas skull width (SKW) was the transverse distance between the centers of the joints between the quadrate and supratemporal bones.

**Fig. 3** Methods for determining contributions to maximal gape area and orientation of major cranial structures. (**A**) An anterior view of a brown treesnake showing how contributions to gape were determined from pins located at the anatomical landmarks shown in Fig. 2. Gape area was partitioned into portions arising from the: skull (SK), quadrate (Q), lower jaw including the dentary (LJD), and intermandibular soft tissues between the tips of the lower jaw (IM). (**B**-**D**) Schematic diagrams showing the orientation of axes and conventions for determining the two-dimensional orientations of major structures in three orthogonal planes. The quadrate, proximal lower jaw and dentary are abbreviated by Q, LJ, and D, respectively, and for the angles of each structure, the two lowercase letters indicate the plane containing the angle. Angles of 90° indicated that the structure pointed straight down in the anterior (**B**) and lateral (**D**) views, whereas a 90° angle in the dorsal view (**C**) indicated that the structure pointed straight forward. The thick lines in the schematic figures illustrate the stick figures used to summarize the mean orientations and locations of the cranial structures in Fig. 7.

**Fig. 4** Scaling relationships between overall size and maximal gape area (Garea) for Burmese pythons (black) and brown treesnakes (gray). (**A**) Mass versus SVL. (**B**) Maximal gape area versus SVL. (**C**) Maximal gape area versus mass. For a given SVL or mass, Burmese pythons had greater mass and Garea than brown treesnakes, but the magnitude of that interspecific difference was less for a given mass than a given SVL. See Table 1 for regression statistics and Table S2 for ANCOVA results.

**Fig. 5** Scaling relationships between measurements of straight-line distances measured at maximal gape. (**A**) Skull length (SKL) versus SVL. (**B**) Skull width (SKW) versus SVL. (**C**) Quadrate length (QL) versus SVL. (**D**) Length of the jaw including the dentary (LJDL) versus SVL. (**E**) Quadrate length (QL) versus skull length (SKL). The slope of the regression for brown treesnakes was significantly steeper than that of Burmese pythons. (**F**) Length of the jaw including the dentary (LJDL) versus skull length (SKL). Maximal gape area (Garea) versus skull length (**G**) and the combined lengths (ALL) of the left and right jaws and quadrates plus skull width (**H**). For a given SVL the Burmese pythons had significantly longer and wider skulls and longer lower jaws than brown treesnakes. However, even after allowing for the dimensions of the bones contributing to gape, the Burmese pythons still had substantially larger gape than the brown treesnakes (**H**). See Table 1 for regression statistics and Table S2 for ANCOVA results.

**Fig. 6** Mean values (+ SE) of the relative contributions to maximal gape area of major cranial structures. SK, Q, LJD, and IM indicate the contributions of skull width, the quadrate bone, the entire lower jaw and the intermandibular skin and soft tissues, respectively (Fig. 3A). Values of IM of the Burmese pythons were more than twice those of brown treesnakes, whereas all remaining structures of the brown treesnakes accounted for greater fractions of Garea than the homologous structures of Burmese pythons.

**Fig. 7** Mean values of angles of major cranial structures at maximal gape and at resting posture as viewed in the transverse, frontal, and sagittal planes. The first and second rows show brown treesnakes at rest (**A**-**C**) and maximal gape (**D**-**F**), respectively, and the third and fourth rows show Burmese pythons at maximal gape (**G**-**I**) and at rest (**J**-**L**), respectively. The dimensions of the figures were standardized for the two species so that the sum of the three-dimensional straight-line lengths of the quadrate (Q), proximal lower jaw (LJ), and dentary (D) were a constant. Within each row images were aligned vertically by the location of the proximal end of the quadrate, and this landmark was also used to align the lateral view images horizontally among all rows. Compared to the brown treesnakes at maximal gape, the Q, LJ, and D of Burmese pythons were more nearly confined to a transverse plane, and the distal ends of these structure were located more laterally relative to their proximal ends. See Table 2 for statistical comparisons between species.

**Fig. 8** Scaling of mass versus cross-sectional area of potential vertebrate prey items for snakes. For a given cross-sectional area, chickens have a much smaller mass than that of the other taxa. See Table 3 for regressions and Table S3 for ANCOVAs comparing species.

**Fig. 9** Maximal relative prey masses (RPM) predicted when relative prey areas equal maximal gape area (RPA = 100%). For both study species and all prey types, scaling relationships (Tables 1, 3) predicted a rapid non-linear decrease in RPM with increased overall snake size. For a given prey type with RPA = 100%, values of RPM for Burmese pythons always exceeded those of brown treesnakes for either a given snake length or snake mass.

**Fig. 10** The predicted effects of prey size relative to gape (RPA) on mass of the prey relative to that of the snake (RPM). For both study species and all prey types, scaling relationships (Tables 1, 3) predicted a curvilinear increase in RPM with increased RPA. Hence, the benefit for RPM from a given incremental increase in RPA was greater for a larger compared to a smaller value of RPA, and decreased snake size (panels **C** versus **A** and **D** versus **B**) exaggerated these differences as well as the differences among different prey types. In panel **A** the values not shown for RPM when RPA = 100% were 154%, 152%, 117%, and 112% for iguanas, alligators, rats, and chickens, respectively. Some biologically unrealistic values of prey size (e.g. in panel **B**, alligators and rabbits are rarely < 40 g, and in panel **C** rats are rarely > 1 kg), were included only to demonstrate the effects of different prey shapes. See Table S4 for additional details.
